# Returning individual research results to participants: Values, preferences, and expectations

**DOI:** 10.1017/cts.2024.568

**Published:** 2024-09-18

**Authors:** Denise A. Kent, Michelle Villegas-Downs, Marina Del Rios, Michael Freedman, Jerry A. Krishnan, Martha G. Menchaca, Crystal L. Patil, Jenny Sculley, Nathan Tintle, Lynn B. Gerald

**Affiliations:** 1 Department of Biobehavioral Nursing Science, University of Illinois Chicago, Chicago, IL, USA; 2 Breathe Chicago Center, Department of Medicine, Division of Pulmonary, Critical Care, Sleep and Allergy, University of Illinois Chicago, Chicago, IL, USA; 3 Department of Human Development Nursing Science, University of Illinois Chicago, Chicago, IL, USA; 4 Department of Emergency Medicine, University of Iowa Hospitals & Clinics, Iowa City, IA, USA; 5 Office of Population Health Sciences, Office of the Vice Chancellor of Health Affairs, University of Illinois Chicago, Chicago, IL, USA; 6 Department of Radiology, University of Illinois Chicago College of Medicine, Chicago, IL, USA; 7 Department of Health Behavior and Biological Sciences, University of Michigan School of Nursing, Ann Arbor, MI, USA; 8 Department of Population Health Nursing Science, University of Illinois Chicago, Chicago, IL, USA

**Keywords:** Return of individual research results, recruitment, retention, engagement research benefits, trust in research, participant-centered research

## Abstract

**Background/objective:**

Disclosing individual research results to participants is not standard practice. The return of individual research results to participants may increase recruitment, retention, and engagement in research. This study’s objective was to explore the preferences, expectations, and experiences of research participants receiving individual research results.

**Methods:**

A mixed-methods approach, consisting of semi-structured interviews and a health literacy assessment, was used with participants enrolled in a cohort study. The interviews were analyzed to produce an understanding of current experiences. Using descriptive analyses, responses were compared to identify alignments and divergences among participants.

**Results:**

Forty-three English-speaking and 16 Spanish-speaking participants enrolled. Ninety-eight percent of participants wanted to receive their individual research results. Seventy-five percent of participants reported they shared results with their healthcare providers. More participants aged 18–65 reported the need to follow up with their provider (70%) as compared to participants > 65 (20%). Two-thirds of participants reported a positive experience receiving their research results; however, 22% reported anxiety and worry. Most participants (69%) described the electronic medical record (EMR) as their preferred method for receiving their results. Yet only 50% of Spanish speakers preferred receiving research results through the EMR compared to 77% of English speakers. Participants with low health literacy preferred receiving study results in person or by phone.

**Conclusion:**

Research participants value receiving their individual research results, and this may increase recruitment and retention within the research enterprise. While more research is needed, the lessons learned from this study lay the groundwork for developing best practices and policies around the return of individual research results.

## Introduction

In the USA, clinical research has been described as fragmented, slow, and costly due in large part to inadequate enrollment of participants [1]. Studies suggest that about 4 in 10 clinical research sites in the USA have inadequate numbers of volunteers enrolled in their studies and an additional 1 in 10 research sites fail to enroll any participants which leads to study termination [2,3]. Recruitment and retention are ubiquitous challenges in clinical research, and the problem is magnified by the lack of representation of people from minority communities [4]. The relatively low participation of minorities in medical research can be partially attributed to a well-founded skepticism toward the medical establishment. Mistrust due to historical, social, and/or political experiences is the most significant factor for low representation of minority groups in research [5]; however, diversity in research is needed to advance science [6]. Inadequate representation may reduce the generalizability of research findings, slow the uptake of proven-effective interventions in communities underrepresented in biomedical research, and widen health inequities.

To address this underrepresentation of minorities in research, the American Thoracic Society and other groups have published consensus papers to examine barriers and recommended interventions to improve recruitment and retention of racial and ethnic minorities for clinical research [4,7]. While strategies such as treating participants with respect, being considerate of participants’ time, and overcoming barriers to retention (such as transportation and childcare) are frequently considered, the return of research results to participants is rarely discussed. Recent literature suggests that the return of research results is one strategy to consider for increasing recruitment, retention, and engagement within the research process [8].

The Office of Human Research Protection (OHRP) identified four aspects of returning research results: (1) return of incidental findings to subjects, (2) return of individual study results to subjects, (3) return of general study results to subjects, and (4) public release of study data. In this recommendation, the return of individual results is considered a separate activity from the return of general study results. Individual research results are results from medical tests, procedures, or questionnaires generated during a study to answer the research question or otherwise support the study (e.g., to determine eligibility) that are specific to one participant [9]. Individual research results are different from aggregate research results that are typically reported at the end of the study to the scientific community and to the general public [9].

The return of individual research results has been shown to increase recruitment, retention, and engagement in the research process [7]. Yet most research participants report not receiving their individual research results. In a study of 3,400 ResearchMatch participants, only 33% reported receiving their individual study results, and 52% reported never being asked if they would like to receive their results [10]. Long *et al*. surveyed health researchers from more than 40 academic medical centers to capture their experiences sharing individual research results with participants [11]. Most researchers (64.5%) believed that individual study results should always be shared with participants, yet only 40% of these researchers reported participating in a study where individual research results were returned to participants. The major reason for not returning these results were due to logistical barriers with sharing individual research results [11]. Nevertheless, reports from the OHRP, Department of Health and Human Services, and the National Academies of Sciences, Engineering, and Medicine (NASEM) conclude that returning valid and actionable individual research results is the ethical responsibility of research teams [9,12]. The 2018 NASEM report cited the lack of evidence to inform best practices for the return of individual research results as a critical need and called on researchers, Institutional Review Boards (IRBs), funding agencies, and policymakers to act. While it is becoming clear that research participants want and benefit from receiving their individual research results, they are inconsistent at all, to participants [13–15]. Therefore, the objective of the **M**y **I**LLInet **R**etu**R**n **O**f **R**esults (**MIRROR**) study was to determine the preferences, expectations, and experiences of research participants receiving individual research results while actively enrolled in a National Institutes of Health (NIH) funded research study on long COVID.

## Materials and methods

### Population

The University of Illinois Chicago (UIC)-led Illinois Research Network (ILLInet) is collaborating on a multicenter study about long COVID [16]. The UIC-ILLInet long COVID study has enrolled more than 750 participants from the Chicago metro area and will obtain hundreds of individual research results (e.g., lab studies, radiology tests, surveys, and procedures), over the course of the four-year study.

The ILLInet site is returning individual research results through the electronic health record (EHR) and/or by phone and mail. If participants choose, their individual research results are also shared with their healthcare provider. MIRROR had the unique opportunity to speak directly with study participants, many of whom were members of racial and ethnic groups (including Spanish-only speakers) traditionally underrepresented in research. Our goal was to recruit a diverse sample of participants who are representative of the race, ethnicity, age, and gender of the ILLInet study population. Additionally, we included 25% Spanish speakers, to enhance our understanding from the perspective of the non-English-speaking participant. We aimed to enroll 60 participants over a four-month period. All study participants provided written informed consent using procedures approved by the UIC IRB (study protocol no. 2022-1196).

### Research design

This descriptive study used a mixed-methods approach, consisting of semi-structured interview questions and a health literacy assessment. To promote rigor and trustworthiness, study procedures and analysis adhered to the qualitative research guidelines set forth by the Consolidated Criteria for Reporting Qualitative Research (COREQ).

### Recruitment

Participants were recruited during an in-person study visit by researchers (DAK and MVD). Study coordinators provided a MIRROR study flyer to participants. A MIRROR investigator then approached participants to determine interest and eligibility. Participants were either interviewed directly after their study visit or were scheduled for an in-person interview at a future date and were compensated $50 for their time.

### Eligibility criteria

Participants were required to meet two inclusion criteria: (1) be enrolled in the UIC-ILLInet study in Chicago and (2) have completed at least the baseline visit (>5 days before enrolling in MIRROR). We recruited participants based on four subgroups established by the long COVID study protocol that included gender, age, ethnicity/race, and language. Within each subgroup, we aimed to have at least six participants in each group (male, female, 18–65 years, > 65 years, white, black, Asian, Hispanic, English, and Spanish-only speakers).

### Data collection procedures

Interview questions were generated based on a review of the literature [17–19]. Of primary importance was hearing directly from research participants about (1) the value and experiences of receiving individual research results, (2) result delivery preference (e.g., MyChart, phone, mail, in person, not at all), and (3) how useful their research results were. The interviews were conducted in person at UIC using the interview guide (Supplementary Material 1: English and Supplementary Material 2: Spanish) and took approximately 30 minutes to complete. The interview included a series of open-ended questions and probes. Interview responses were reviewed after approximately 40% were completed to determine if the responses provided answered our research questions. One question was added to specifically assess what participants were learning from their individual research results.

Following the interviews, a health literacy assessment was completed using the Short Assessment of Health Literacy (SAHL) [20]. The SAHL has an English and Spanish version with good reliability (0.89 English and 0.80 Spanish) [20]. For the SAHL, the researcher presents 18 test terms on a flashcard, one at a time. Participants are asked to read the test term aloud and choose the word closest in meaning. The SAHL tests the participant’s comprehension, as well as pronunciation of health-related terms, and takes 2–3 minutes to administer. A score of 15–18 is considered adequate health literacy.

Interviews were recorded using Otter Ai (https://otter.ai/). English interviews were recorded and transcribed in real time. Spanish interviews were recorded in Otter Ai but transcribed using Sonix (https://sonix.ai/) as Otter Ai does not have the capability to transcribe Spanish speech. Transcripts were verified by a member of the research team to ensure accuracy between the audio and text transcription. Transcripts were then uploaded to Dedoose (https://www.dedoose.com/), for analysis.

### Analysis

Descriptive statistics were used to summarize demographic characteristics and the SAHL. A codebook was created to guide researchers in categorizing the participant’s responses to ensure consistency and transparency in the process. An experienced qualitative researcher guided the data analysis process. Each English transcript was coded by either DAK or MVD, and each Spanish transcript was coded by either MVD or MRD both biliterate native Spanish speakers. Spanish transcripts were thematically analyzed in their original language. A random set of six English-speaking participants were coded by DAK and MVD, and a random set of three Spanish-speaking transcripts were coded by both MVD and MRD. Percent agreement was used to ensure the reliability of the coding process across individuals. The percent agreement was calculated at 97% for English transcripts and 95% for Spanish transcripts. Thematic analysis was achieved when no new codes or themes were generated during data analysis.

## Results

### Participant characteristics

Sixty interviews were completed between November 2, 2022, and February 28, 2023. One participant was interviewed twice, but only the first interview was retained for analysis, providing a total sample size of 59. Most interviews were conducted in English, and a quarter were conducted in the participant’s only language, Spanish. The response rate for English-speaking participants was 84% (43/51) and for Spanish-speaking participants was 80% (16/20). Summaries for health literacy scores, mean age, and demographic data are reported in Table 1. Themes were compared across demographic subgroups (age, gender, race/ethnicity, health literacy, and primary language); however, few differences were observed across demographic characteristics (Supplementary Material 3).

**Table 1.** Demographic characteristics of MIRROR sample

Characteristics	Total (*n* = 59)	English (*n* = 43)	Spanish (*n* = 16)
Age: Mean (SD)	51.2 years (13.1)	50.6	51.5
18–65 years	47 (80%)	35	11
>65 years	12 (20%)	8	5
Gender			
Female	35 (59%)	26	9
Male	24 (41%)	17	7
Ethnicity/race			
Asian (%)	3 (5%)	3	0
Black (%)	17 (29%)	17	0
Hispanic (%)	23 (39%)	7	16
White (%)	16 (27%)	16	0
Education			
High school (HS) or less	16 (65%)	9	7
Some college or post-HS training	18 (31%)	11	7
Bachelor’s degree	13 (22%)	12	1
Graduate degree	12 (20%)	11	1
Health literacy: Mean (SD)	16.5 (1.7)	16.5 (1.8)	16.8 (1.2)
Adequate (≥15)	52 (88%)	37	15
Low (<15)	7 (12%)	6	1

### Interview responses

#### Why are you participating in the ILLInet study?

Nearly all participants shared an altruistic desire to enroll in the ILLInet study. Some participants shared near-death experiences with COVID-19, while others reported devastating effects on their family and friends. Overall participants stated they were hopeful the study could provide answers about COVID-19.

(E: refers to English speaker and S: refers to Spanish speaker)

**Subject 54-E** *I wanted to join just because I’ve had COVID twice. And I’ve had side effects and I’ve heard of different people having different side effects and then you see stuff online and just from talking to other people about things they are experiencing it is different. So, I think the study’s aiming to look at why that is and I really want to know… if I can help in any way, I will do it.*

**Subject 6-E** *My brother has dealt with a lot of post COVID effects when he got it, back when the pandemic first started, in like April or May. So, when I heard about it, I’ve never had COVID and they needed controls….I will gladly do it.*

Most participants reported a desire to receive their individual study results, and almost half of the participants indicated that receiving their results was a powerful motivator for participating in the study. More than half of the participants reported having unanswered questions related to COVID-19 that their healthcare providers could not answer.

**Subject 11-E** *Because there’s a lot of things that happened to me after COVID, umm, that I would like other people to know about. I had a lot of things that still, um, how can I say that, lingering after the COVID that, umm, still going on in my body, so I do not mind sharing it. And if they have a study, I would rather you guys know what’s going on. And it could help…so many different things to happen to so many different people.*

#### What did you learn from receiving your ILLInet research results?

The ILLInet study returned individual research results to its participants and provided the option for those results to be shared directly with their primary care team. Table 2 indicates the majority of participants reported learning health information that was useful. Almost three-quarters of participants between 18 and 65 years old reported they needed to follow up with their primary care provider (PCP) based on their study results; however, only 20% of those > 65 years old reported a need to follow up with their PCP. After consultation with their PCP, three individuals received treatment for medical concerns identified through their participation in the study.

**Table 2.** What did you learn from your results?

	Total	Language		Age	
	*N* (%)	English	Spanish	18–65 years	>65 years
Number of participants*	32	21	11	27	5
Health information (# responses)	28 (88%)	18 (86%)	10 (91%)	24 (89%)	4 (80%)
Need to see provider (# responses)	10 (3%)	9 (43%)	1 (9%)	19 (70%)	1 (20%)
Nothing (# responses)	2 (6%)	1 (5%)	1 (9%)	1 (4%)	1 (20%)
Validation of management plan (# responses)	2 (6%)	2 (10%)	0	2 (7%)	0

*N = 32 instead of 59 because this question was added midway through data collection, hence was not asked to all participants.

**Subject 50-E** *I remember one [result] specifically, with Vitamin D. My levels came back low and so they [research nurse] asked me to consult with my primary care, which I did. And my doctor gave me something for it [low vitamin D result] and it was solved.*

Other participants describe their results as an added benefit to participating in research.

**Subject 34-E** *It’s been great. I like having all my results in there [MyChart]. I think that was a really big perk along with the study, to be able to actually see your lab results. So, I love it.*

**Subject 46-S** *English Translation: Actually, yes, I have learned. I mean, [in] urine there are certain numbers that I need to be balanced. So, if they are too high, it means that I have an infection or that there is something abnormal. So, the truth is, that I have learned a lot because every time I get an abnormal result [I] investigate to see what the consequences of those abnormal [results] might be.*

#### What is your preferred method for receiving your results?

MyChart, the EHR associated with the ILLInet group, allows individuals to receive their research results (https://www.mychart.org). Table 3 shows that the majority of participants prefer MyChart for receiving their individual research results. When examining preferred methods by language, almost half of Spanish speakers preferred to receive results via phone or mail, while most of the English speakers preferred to receive their results via MyChart. Those with low health literacy reported a preference for receiving study results in person or by phone.

**Table 3.** Preferred way to receive study results?

	Total	Language		Age	
	*N* (%)	English	Spanish	18–65 years	>65 years
Number of participants*	59	43	16	49	10
MyChart	41 (69%)	33 (77%)	8 (50%)	36 (73%)	5 (50%)
Email	5 (8%)	3 (7%)	2 (13%)	5 (10%)	0
In person	7 (12%)	6 (14%)	1 (6%)	7 (14%)	0
Mailed letter	5 (8%)	1 (2%)	4 (25%)	2 (29%)	3 (30%)
Phone call	9 (15%)	6 (14%)	3 (19%)	8 (16%)	1 (10%)
Text	3 (5%)	3 (7%)	0	3 (6%)	0
Other	1(2%)	1(2%)	0	0	1 (10%)

*More than 1 response possible therefore total number of responses > than *N*.

#### What was your experience like receiving your research results?

Most participants described their experience receiving their individual research results through MyChart as easy, timely, and accessible (coded as positive). However, this was not the experience for all participants. Almost a fourth of participants reported no experience with MyChart, while 19% reported improvements are needed (reported in Table 4). Only half of Spanish speakers report a positive experience in contrast to 72% of English speakers. A similar difference was noted in responses stating no experience with MyChart.

**Table 4.** Experience receiving test results in MyChart?

	Total	Language		Age	
	*N* (%)	English	Spanish	18–65 years	>65 years
Number of participants	59	43	16	49	10
Positive experience	39 (66%)	31 (72%)	8 (50%)	34 (69%)	5 (50%)
Neutral experience	1 (1.7%)	1 (2.3%)	0	1 (2%)	0
Needs improvement	11 (19%)	9 (21%)	2 (13%)	9 (18%)	2 (20%)
No experience	14 (24%)	8 (19%)	6 (38%)	10 (2%)	4 (40%)

**Subject 32-E** *I loved it, except that, the only thing I have a problem with is that you have to ask for a code to get into MyChart. I can never get that code. [The study coordinator] told me to contact the help desk. I do not do very well with that kind of stuff. I’m not very patient with not having access to that kind of stuff, my stuff so I didn’t do that. I got angry.*

**Subject 43-S** *English Translation: I entered my password, but it told me it was not the [correct] password. So what I did is, ask for the password. It sent me a password and all the information [to log in to MyChart]. I put in that information, but it wouldn’t let me in [access]. It [MyChart message] tells me the username doesn’t match. Right now, I cannot see the results, if they can fix the application, so that they can send me the correct password.*

#### Any disadvantages to receiving your individual research results?

While most participants reported no disadvantages to receiving their individual study results (Table 5), 40% reported: worry and anxiety, concerns about security and privacy, as well as uncertainty about the meaning of their results. One participant shared their experience of receiving research results late on a Friday afternoon and then having to wait until Monday to contact their doctor about what their results may mean. Others report worry about what their results mean for their employment and school.

**Table 5.** Any disadvantages to receiving your results?

	Total	Language		Age	
	*N* (%)	English	Spanish	18–65 years	>65 years
Number of participants	55	39	16	46	9
No disadvantages	33 (60%)	21 (54%)	11 (69%)	27 (59%)	5 (56%)
Worry/anxiety	12 (22%)	10 (26%)	2 (13%)	9 (20%)	3 (33%)
Not receiving all results	2 (4%)	1 (3%)	1 (6%)	2 (4%)	0
Receiving abnormal results	6 (11%)	5 (13%)	1 (6%)	5 (11%)	1 (11%)
Security/privacy concerns	4 (7%)	3 (8%)	1 (6%)	3 (7%)	1 (11%)
Uncertainty about what results mean	6 (11%)	6 (15%)	0	5 (11%)	1 (11%)
Other	4 (7%)	3 (8%)	1 (6%)	4 (9%)	0

**Subject 55-E** *That’s where it gets to be kind of dangerous for people like me, who are immediately going to grab that data and take a dive. And possibly freak themselves out, which I did last time. Some of the tests that I did not recognize, I said well what is this for? Well, it turns out that was because of the steroid injections [I had for my knees the day before my last blood draw]. That never occurred to me. So here I am completely losing it.*

**Subject 42-E** *I could receive information saying, here are the results, but what [do] these results mean? I think it’s still an advantage of having them in your hands, [but it’s] somewhat of a disadvantage, if you do not have the information of what’s triggered…And here’s the next step that we’re going to take. You guys put it up on MyChart, and then that’s it. It can be [an] abnormal reading…but there’s no follow-up saying, go do this, or this was abnormal. Here’s why*.

**Subject 28-E** *I guess just privacy issues. I wouldn’t want it to, like affect employment or, like education or anything like that. I mean, you know, they say that it doesn’t but it’s such a subjective thing that you cannot really prove one way or another*.

#### Did you share your research results with anyone?

Participants are encouraged to share their research results with their healthcare providers so they may interpret the results within the context of the participants’ overall health. Most MIRROR participants report sharing their research results with PCPs. A higher percentage of English speakers shared their results with their PCP when compared to Spanish speakers (Table 6).

**Table 6.** Did you share your results?

	Total	Language		Age	
	*N* (%)	English	Spanish	18–65 years	>65 years
Yes	52 (90%)	42 (98%)	11 (69%)	45 (92%)	7 (75%)
PCP (primary care provider) (# responses)	39 (75%)	34 (81%)	5 (50%)	33 (73%)	6 (86%)
Spouse, children, parents (# responses)	37 (71%)	30 (71%)	7 (70%)	32 (71%)	5 (71%)
Extended family and/or friends (# responses)	15 (29%)	15 (36%)	0	11 (24%)	4 (57%)
Community or religious leaders (# responses)	1 (2%)	1 (2%)	0	0	1 (17%)
No	6 (10%)	1 (2%)	5 (31%)	4 (9%)	2 (29%)

**Subject 28-E** *I’ve accessed my blood test results and those were helpful for my primary care physician. There were a couple of things that were a little bit low like my good cholesterol was low and my vitamin D levels were normal but low and she just suggested a couple of things that I could do to bring those up.*

**Subject 62-S** *English translation: I told my main doctor, Dr C. I’m getting tests [done for a study]. This is what is happening and he told me that it is very good*.

### Scenario 1 and 2 responses

Participants were asked to reflect on two scenarios during the interview. Both scenarios present an abnormal result that indicates their life might be cut short. In scenario 2, we specify no cure or treatment is available. Nearly all participants responded they would want to receive the results in both scenarios.

## Discussion

This study had four major findings: (1) Participants value receiving their individual research results. (2) Participants are acting on their individual research results. (3) Individual research results may be linked to anxiety and worry. (4) Research teams need to tailor the return of results based on participant preferences.

### Participants value receiving their individual research results

ILLInet study participants not only value receiving their individual research results, but they are also using this information, with input from their healthcare providers, to improve their health. Participants shared that receiving their individual lab results, along with follow-up from a research nurse when results were abnormal, was an unexpected benefit. Some participants commented they appreciated the effort the ILLInet study team made to ensure that their research results were returned to them and their PCP. Participants reported wanting their individual research results even when the abnormal test result would indicate a decrease in life expectancy and there is no treatment available. MIRROR’s results are consistent with other published studies [13,14] that report research participants’ value receiving their individual research results, but, to our knowledge, this is the first study to report research participants are using their results to improve their health. A lack of transparency between research teams and participants may be leading to lower than desired retention rates. Specific insights gleaned through MIRROR interviews highlight participants’ desire to participate in research to benefit society and themselves.

### Participants are acting on their individual research results

Many adults (73%) between 18 and 65 years old reported acting on their research results, such as following up with their PCP, while only 20% of adults over 65 years of age reported the need to follow up with their provider. This variation between younger (18–65 years) and older (>65 years) participants may be due to the likelihood that older adults are already seen regularly by their PCP for chronic conditions. For younger adults who are not engaged regularly with a provider, research may offer valuable individual health information. Research teams need to consider this benefit to potential participants when recruiting younger adults to participate in research.

### Individual research results may be linked to anxiety and worry

Most participants reported a positive experience with receiving their individual research results; however, worry and anxiety were reported among 22% of participants. Some participants expressed uncertainty about receiving individual laboratory results via the EHR without any explanation. Participants also stated they were unsure whether they should follow up with their PCP. Researchers are often limited in their ability to provide comprehensive interpretations of research results for participants since research tests are not completed for medical purposes. Researchers should consider how they can return results in a timely manner, while appropriately contextualizing research results to minimize excessive worry and encourage appropriate medical follow-up. More work is needed to address these issues and develop best practices.

### Research teams need to tailor the return of results based on preferences

While MyChart was the preferred method for receiving results among participants, a difference was noted when examining the results by language. Half of Spanish-speakers prefer an alternative method for receiving their results. Among all participants, 30% stated receiving their research results in-person or via mail or phone was preferred. Participants’ experiences with MyChart were generally positive, yet some voiced barriers to access or reported no familiarity with it at all, as was the case for 38% of Spanish speakers. Most participants reported having access to the internet; however, the type of internet device used varied. The small size of a smartphone screen compared with a computer or iPad screen may be a barrier for some to view their results. Receiving a call from a study nurse to help interpret abnormal results was viewed as an important complement to sharing research results through MyChart. When returning individual research results, research teams need to consider customizing methods based on individual preferences. Delivering a tailored research experience may be difficult for research teams that may have limited financial resources, staffing, and planning periods. Fields like implementation science and human-centered design offer theories, frameworks, methods, and structured processes that can help researchers return results in ways that are desirable to participants and feasible to implement [21–23].

### Strengths and limitations

The MIRROR study had several notable strengths: (1) All interviews were conducted by one of two nurse clinicians (DAK and MVD). (2) Spanish interviews were conducted by MVD a native biliterate Spanish speaker to optimize language concordance. (3) MIRROR recruited a racially and ethnically diverse sample that was representative of the racial and ethnic diversity of the parent study enrolled at the UIC ILLInet. (4) Health literacy data were obtained on each participant.

Semi-structured interviews are a preferred method for exploring participant thoughts, feelings, and beliefs about a particular topic; however, there are limitations. For example, an interview can only assess a select number of concepts before the participant fatigues from the interview. Another limitation is that our sample had an overrepresentation of females and consisted of individuals with adequate health literacy. Finally, MIRROR was recruited from a single research site in Chicago and is limited to describing participants’ experiences with one EHR, MyChart, within one NIH study. More studies are needed to explore the return of research results focusing on alternative EHR platforms and among low health literacy and rural populations.

### Implications

While the findings of the MIRROR study are specific to urban minority participants in an NIH-funded research project, MIRROR offers an in-depth understanding of what research participants’ experiences, expectations, and preferences are around the return of individual research results. This study highlights the desire research participants have to receive their individual research results even when it cannot improve their health, as was learned from scenarios 1 and 2. Fostering a more collaborative and participant-centered approach through the return of individual research results may increase recruitment, retention, and engagement in research. This may be particularly impactful in improving the recruitment and retention of minority populations in research. More research is needed to establish best practices and to determine optimal methods for returning individual research results to participants. In doing so, we as a research community endeavor to ensure participants are not only valued contributors but also direct beneficiaries of the research process.

## Conclusion

The importance of research participants is undeniable, and addressing their needs and preferences is vital to enhancing the collaborative relationship between researchers and volunteers. The issue of inadequate enrollment in clinical research studies in the USA poses a significant challenge. The underrepresentation of people from minority racial and ethnic backgrounds also impedes the generalizability and effectiveness of research findings. The need for a more inclusive and diverse participant pool is evident and remains a crucial factor in health inequities experienced by underrepresented minority groups in research. The return of individual research results to participants can be a promising way to increase recruitment, retention, and engagement within the research process. From this study, we learned that MIRROR participants value receiving their individual research results and are using this information to improve their health. Returning individual research results to participants also served as a powerful motivator for their participation in research. The MIRROR study conducted within the UIC-ILLInet initiative provides valuable insights into the preferences, expectations, and experiences of research participants, shedding light on their eagerness to engage in research in a more collaborative and transparent manner. While more research is needed, the results learned from this study lay the groundwork for developing best practices and policies around the return of individual research results.

## Supporting information

Kent et al. supplementary material 1 Kent et al. supplementary material

Kent et al. supplementary material 2 Kent et al. supplementary material

Kent et al. supplementary material 3 Kent et al. supplementary material

## Supplementary material

The supplementary material for this article can be found at https://doi.org/10.1017/cts.2024.568.
