# Supplementary material for: Returning individual research results to participants: Values, preferences, and expectations
Source: J Clin Transl Sci. 2024 Sep 18;8(1):e126. doi: 10.1017/cts.2024.568 (PMC11428116; doi:10.1017/cts.2024.568)
Supplement: Kent et al. supplementary material 1 — Kent et al. supplementary material [file S2059866124005685sup001.docx]

**A1: English Interview Guide**

**Introduction**

Thank you for volunteering. Our goal for this interview is to learn about your preferences and expectations regarding the return of your study results. We will be recording this interview. Know that we are interested in your thoughts and opinion –we are interested in what you have to say; in other words, there are no right or wrong answers. Before we start, do you have any questions for me?

I would like to begin by defining two terms:

- *Individual study results*: your personal health information
- *Aggregate study results*: the findings or results learned from the study as a whole.

If you need me to define these terms at another time, please let me know.

1. Tell me about why you decided to participate in the RECOVER study
   1. What do you hope to learn?
2. Tell me about how you use the internet (phone, wifi at home, work)?
   1. Where do you access the internet? Is your access reliable?
   2. What do you use the internet for? Health information?
      1. What about MyChart? Tell me about your experience with MyChart.
3. The RECOVER study currently makes your results available to you in My Chart.
   1. Tell me about your experience with the RECOVER study and MyChart so far?
   2. Is there any other way you would want to get this information?

TRANSITION STATEMENT: As you know, the RECOVER study is collecting a lot of data about you and your health. If fact, you will be getting lots of personal health information/results from this study.

1. Can you tell me about the various types of health information that are being collected as part of the RECOVER study?
2. What kind of health information will be shared with you?
   1. In RECOVER, you will get laboratory results back from blood draws. Have you gotten these results back?
      1. If so, tell me about how you got this information.
      2. What did you learn from these results?
      3. How do you feel about these results being available in My Chart?
   2. In RECOVER, you participate in physical exams. Have you gotten these results back?
      1. If, no would you like to receive?
      2. If so, tell me about how you got this information.
      3. What did you learn from these results?
      4. How do you feel about these results being available in My Chart?
   3. In RECOVER, they collect mental health information. Have you gotten these results back?
      1. If no, would you like to receive those?
      2. If so, tell me about how you got this information.
      3. What did you learn from these results?
      4. How do you feel about these results being available in My Chart?
   4. In RECOVER, you might get Xrays, CTs, or MRIs. Have you gotten these results back?
      1. If so, tell me about how you got this information.
      2. What did you learn from these results?
      3. How do you feel about these results being available in My Chart?
3. How important are these results to you?
   1. What do these results mean to you?
   2. Is there a downside to getting these results?
4. Have you shared your results with anyone else?
   1. What about your primary care provider?
      1. Do you worry about your results being shared with anyone?

Let’s run through a few scenarios:

1. Imagine a situation in which an abnormal result would indicate, for example, that on average your life might be cut short? Would this be a result that you would want to know? Why or why not?
2. Now imagine that this same abnormal result could not be used by you or your medical provider to make changes to your medical care because, for example, no known treatments exist. Would that affect whether you would want to know about this result?

We are nearing the end of our interview, and this is all good information you are sharing.

1. Can you summarize what the advantages are if any of receiving your study results? Any disadvantages?
2. Through the RECOVER study currently you have your results available to you in My Chart. Is this your preferred way to receive study results (text message, phone, email, in person, mail, telehealth)?
3. Currently, with the RECOVER study your results are available in My Chart in real time or in other words when they are available, would you like to receive study results when they are available as it currently is or when they are all available, weekly, monthly, every 3 mos. every 6 mos. or never?

**Closing by Interviewer to Participant**

This is all good information you are sharing. As we wrap up the interview is there anything else you think is important for me to know about you receiving your study results?

Thank you for participating in this interview.
