# Supplementary material for: Returning individual research results to participants: Values, preferences, and expectations
Source: J Clin Transl Sci. 2024 Sep 18;8(1):e126. doi: 10.1017/cts.2024.568 (PMC11428116; doi:10.1017/cts.2024.568)
Supplement: Kent et al. supplementary material 2 — Kent et al. supplementary material [file S2059866124005685sup002.docx]

**A-2**: **Spanish Interview Guide**

**Introducción**

Gracias por ser voluntario. Nuestro objetivo para esta entrevista es conocer sus preferencias y expectativas con respecto a la devolución de sus resultados del estudio. Esta entrevista será grabada. Sepa que estamos interesados ​​en sus pensamientos y opiniones; estamos interesados ​​en lo que tiene que decir; en otras palabras, no hay respuestas correctas o incorrectas. Antes de comenzar, ¿tiene alguna pregunta para mí?

Me gustaría comenzar definiendo dos términos:

1. *Resultados de estudios individuales*: su información de salud personal
2. *Resultados agregados del estudio*: los hallazgos o resultados aprendidos del estudio en su totalidad.

Si necesita que defina estos términos en otro momento, hágamelo saber.

1. Cuéntame por qué decidiste participar en el estudio RECOVER.
2. ¿Qué esperas aprender?
3. Cuéntame cómo usas la Internet (teléfono, wifi en casa, trabajo).
4. ¿Dónde accedes la Internet? ¿Su acceso es confiable?
5. ¿Para qué usas la internet? ¿Información de salud?
6. ¿Qué tal MyChart? Cuéntame sobre tu experiencia con MyChart.
7. Actualmente, el estudio RECOVER pone sus resultados a su disposición en MyChart.
8. Cuéntame sobre tu experiencia con el estudio RECOVER y MyChart hasta ahora.
9. ¿Hay alguna otra forma en la que le gustaría obtener esta información?

DECLARACIÓN DE TRANSICIÓN: Como sabe, el estudio RECOVER está recopilando muchos datos sobre usted y su salud. De hecho, recibirá mucha información/resultados de su salud personal en este estudio.

1. ¿Puede decirme sobre los distintos tipos de información de salud que se están colectando como parte del estudio RECOVER?
2. ¿Qué tipo de información de salud se compartirá con usted?
3. Como parte del estudio RECOVER, recibirá resultados de laboratorio de las extracciones de sangre. ¿Ha recibido estos resultados?
4. Si ha recibido resultados de laboratorio, cuénteme cómo recibió esta información.
5. ¿Qué aprendiste de estos resultados?
6. ¿Qué le parece que estos resultados estén disponibles en MyChart?
7. En RECOVER, participas en exámenes físicos. ¿Has obtenido estos resultados?
8. En caso negativo: ¿te gustaría recibirlos?
9. Si ha recibido resultados de los exámenes físicos, cuénteme cómo recibió esta información.
10. ¿Qué aprendiste de estos resultados?
11. ¿Qué le parece que estos resultados estén disponibles en My Chart?
12. En RECOVER, recopilan información sobre su salud mental. ¿Has recibido estos resultados?
13. En caso negativo: ¿te gustaría recibirlos?
14. Si ha recibido resultados de su salud mental, cuénteme cómo recibió esta información.
15. ¿Qué aprendiste de estos resultados?
16. ¿Qué le parece que estos resultados estén disponibles en My Chart?
17. En RECOVER, es posible que le hagan radiografías, tomografías computarizadas o resonancias magnéticas. ¿Has obtenido estos resultados?
18. Si ha recibido resultados de estos exámenes, cuénteme cómo recibió esta información.
19. ¿Qué aprendiste de estos resultados?
20. ¿Qué le parece que estos resultados estén disponibles en My Chart?
21. ¿Qué importancia tienen estos resultados para usted?
22. ¿Qué significan estos resultados para usted?
23. ¿Existe algún inconveniente en obtener estos resultados?
24. ¿Has compartido tus resultados con alguien más?
25. ¿Qué tal con tu proveedor de atención primaria?
26. ¿Le preocupa que sus resultados del estudio se compartan con alguien más?

Repasemos algunos escenarios:

1. ¿Imagine una situación en la que un resultado anormal indicaría, por ejemplo, que su promedio de vida podría ser reducida? ¿Sería este un resultado que le gustaría saber? ¿Por qué o por qué no?
2. Ahora imagine que ni usted ni su proveedor médico podrían utilizar este mismo resultado anormal para realizar cambios en su atención médica porque, por ejemplo, no existen tratamientos conocidos. ¿Eso afectaría si desearía conocer este resultado?

Nos acercamos al final de nuestra entrevista y toda esta información que está compartiendo es muy buena.

1. ¿Puede resumir cuáles son las ventajas, si las hay, de recibir tus resultados del estudio? ¿Hay desventajas?
2. A través del estudio RECOVER tus resultados están disponibles en MyChart. ¿Es esta tu forma preferida de recibir tus resultados del estudio (mensaje de texto, teléfono, correo electrónico, en persona, correo, telesalud)?
3. Actualmente, tus resultados del estudio RECOVER están disponibles en MyChart en tiempo real, osea cuando estén disponibles. ¿Te gustaría recibir los resultados del estudio cuando estén disponibles como están actualmente o cuando estén todos disponibles, semanalmente, mensualmente, cada 3 meses, cada 6 meses, o nunca?

**Cierre del entrevistador al participante**

Toda la información que está compartiendo es muy buena. Mientras concluimos la entrevista, ¿hay algo más que considere importante que sepa sobre la devolución de los resultados del estudio?

Gracias por participar en esta entrevista.
