# Supplementary material for: Returning individual research results to participants: Values, preferences, and expectations
Source: J Clin Transl Sci. 2024 Sep 18;8(1):e126. doi: 10.1017/cts.2024.568 (PMC11428116; doi:10.1017/cts.2024.568)
Supplement: Kent et al. supplementary material 3 — Kent et al. supplementary material [file S2059866124005685sup003.docx]

**Table A3**

|  | **Gender** | | **Race and Ethnicity** | | | | **Health  Literacy** | |
| --- | --- | --- | --- | --- | --- | --- | --- | --- |
| **Interview Questions** | **Female** | **Male** | **Asian** | **Black** | **Hispanic** | **White** | **Adequate** | **Low ^1^** |
| **1. Preferred way to receive study results? (# participants)** | 34 | 25 | 3 | 18 | 21 | 17 | 52 | 7 |
| MyChart | 26 (76%) | 15 (60%) | 3 (100%) | 9 (50%) | 13 (62%) | 16 (94%) | 38 (73%) | 3 (43%) |
| Email | 5 (15%) | 0 | 1 (33%) | 1 (6%) | 2 (10%) | 1 (7%) | 5 (10%) | 0 |
| In person | 4 (12%) | 3 (12%) | 0 | 5 (28%) | 2 (10%) | 0 | 5 (10%) | 2 (29%) |
| Mailed letter | 3 (9%) | 2 (8%) | 1 (33%) | 0 | 4 (20%) | 0 | 5 (10%) | 0 |
| Phone call | 3 (9%) | 6 (24%) | 0 | 4 (22%) | 4 (20%) | 1 (7%) | 7 (13%) | 2 (29%) |
| Text | 2 (6%) | 1 (4%) | 0 | 2 (11%) | 1 (5%) | 0 | 3 (6%) | 0 |
| Other | 0 | 1(4%) | 0 | 0 | 0 | 1 (7%) | 0 | 1(14%) |
|  |  |  |  |  |  |  |  |  |
| **2. Experience receiving test results in MyChart?**  **(# participants)** | 34 | 25 | 3 | 18 | 21 | 17 | 52 | 7 |
| Positive experience | 25 (74%) | 14 (56%) | 2 (50%) | 10 (56%) | 13 (62%) | 14 (82%) | 36 (69%) | 3 (43%) |
| Neutral experience | 1 (3%) | 0 | 0 | 1 (6%) | 0 | 0 | 1 (2%) | 0 |
| Needs improvement | 7 (21%) | 4 (16%) | 1 (33%) | 2 (11%) | 3 (14%) | 5 (29%) | 9 (17%) | 2 (29%) |
| No experience | 5 (15%) | 9 (36%) | 0 | 8 (44%) | 6 (29%) | 0 | 11 (21%) | 3 (43%) |
|  |  |  |  |  |  |  |  |  |
| **3. What did you learn from your results? (# participants)** | 21 | 11 | 2 | 5 | 14 | 11 | 29 | 3 |
| Health information | 19 (90%) | 9 (82%) | 1 (50%) | 5 (100%) | 13 (93%) | 9 (82%) | 25 (86%) | 3 (100%) |
| Need to see provider | 8 (38%) | 2 (18%) | 1 (50%) | 2 (40%) | 3 (21%) | 4 (36%) | 9 (31%) | 1 (33%) |
| Nothing | 1 (5%) | 1 (9%) | 0 | 2 (40%) | 1 (7%) | 1 (10%) | 2 (7%) | 0 |
| Validation of management plan | 1 (5%) | 1 (9%) | 0 | 0 | 1 (7%) | 1 (10%) | 2 (7%) | 0 |
|  |  |  |  |  |  |  |  |  |
| **4. Did you share your results? (# participants)** | 33 | 25 | 3 | 18 | 20 | 17 | 51 | 7 |
| No | 3 (9%) | 3 (12%) | 0 | 1 (6%) | 5 (25%) | 0 | 5 (10%) | 1 (14%) |
| Yes | 30 (91%) | 22 (88%) | 3 (100%) | 17 (94%) | 15 (75%) | 17 (100%) | 46 (90%) | 6 (86%) |
| **Who did you share your results with?** |  |  |  |  |  |  |  |  |
| With provider | 23 (77%) | 16 (73%) | 2 (67%) | 14 (78%) | 10 (67%) | 13 (76%) | 35 (76%) | 4 (67%) |
| Spouse, children, parents | 21 (70%) | 16 (73%) | 2 (67%) | 13 (72%) | 10 (67%) | 12 (71%) | 32 (70%) | 5 (83%) |
| Extended family and/or friends | 11 (37%) | 4 (18%) | 2 (67%) | 6 (33%) | 1 (7%) | 6 (35%) | 14 (30%) | 1 (17%) |
| Community or religious leaders | 1 (3%) | 0 | 0 | 0 | 0 | 1 (6%) | 1 (2%) | 0 |
|  |  |  |  |  |  |  |  |  |
| **5. Any disadvantages to receiving your results?**  **(# participants)** | 32 | 23 | 3 | 17 | 21 | 14 | 49 | 6 |
| No disadvantages | 17 (53%) | 15 (65%) | 0 | 15 (88%) | 14 (67%) | 3 (21%) | 26 (53%) | 6 (100%) |
| Worry / Anxiety | 9 (28%) | 3 (13%) | 2 (67%) | 1 (6%) | 3 (14%) | 6 (43%) | 12 (24%) | 0 |
| Not receiving all results | 0 | 2 (9%) | 0 | 0 | 1 (5%) | 1 (7%) | 2 (4%) | 0 |
| Receiving abnormal results | 5 (22%) | 1 (4%) | 2 (67%) | 0 | 1 (5%) | 3 (21%) | 6 (12%) | 0 |
| Security / Privacy concerns | 3 (9%) | 1 (4%) | 0 | 0 | 2 (10%) | 2 (14%) | 4 (8%) | 0 |
| Uncertainty about what results mean | 3 (9%) | 3 (13%) | 1 (33%) | 0 | 0 | 5 (36%) | 6 (12%) | 0 |
| Other | 2 (6%) | 2 (9%) | 0 | 2 (12%) | 2 (10%) | 0 | 4 (8%) | 0 |

**For each question participants could provide more than 1 response, therefore the number of responses may exceed the number of participants.**

**^1^Low health literacy defined as score 0 to 14 on 18-item Short Assessment of Health Literacy, validated in English and Spanish.**
